# Clinically Applicable Cyclotron-Produced Gallium-68 Gives High-Yield Radiolabeling of DOTA-Based Tracers

**DOI:** 10.3390/biom11081118

**Published:** 2021-07-29

**Authors:** Emma Jussing, Stefan Milton, Erik Samén, Mohammad Mahdi Moein, Lovisa Bylund, Rimma Axelsson, Jonathan Siikanen, Thuy A. Tran

**Affiliations:** 1 Department of Oncology and Pathology, Karolinska Instituted, SE-171 77 Stockholm, Sweden; stefan.milton@sll.se (S.M.); erik.samen@ki.se (E.S.); mohammad.moein@sll.se (M.M.M.); jonathan.siikanen@ki.se (J.S.); 2 Department of Radiopharmacy, Karolinska University Hospital, SE-171 76 Stockholm, Sweden; Lovisa.bylund@sll.se; 3 Department of Medical Radiation Physics and Nuclear Medicine, Karolinska University Hospital, SE-171 76 Stockholm, Sweden; Rimma.Axelsson@ki.se; 4 Department of Clinical Science, Intervention and Technology, Karolinska Instituted, SE-171 77 Stockholm, Sweden

**Keywords:** cyclotron-produced gallium-68, ascorbate, DOTATOC, FAPI-46, DOTA chelator

## Abstract

By using solid targets in medical cyclotrons, it is possible to produce large amounts of ^68^GaCl_3_. Purification of Ga^3+^ from metal ion impurities is a critical step, as these metals compete with Ga^3+^ in the complexation with different chelators, which negatively affects the radiolabeling yields. In this work, we significantly lowered the level of iron (Fe) impurities by adding ascorbate in the purification, and the resulting ^68^GaCl_3_ could be utilized for high-yield radiolabeling of clinically relevant DOTA-based tracers. ^68^GaCl_3_ was cyclotron-produced and purified with ascorbate added in the wash solutions through the UTEVA resins. The ^68^Ga eluate was analyzed for radionuclidic purity (RNP) by gamma spectroscopy, metal content by ICP-MS, and by titrations with the chelators DOTA, NOTA, and HBED. The ^68^GaCl_3_ eluate was utilized for GMP-radiolabeling of the DOTA-based tracers DOTATOC and FAPI-46 using an automated synthesis module. DOTA chelator titrations gave an apparent molar activity (AMA) of 491 ± 204 GBq/µmol. GMP-compliant syntheses yielded up to 7 GBq/batch [^68^Ga]Ga-DOTATOC and [^68^Ga]Ga-FAPI-46 (radiochemical yield, RCY ~ 60%, corresponding to ten times higher compared to generator-based productions). Full quality control (QC) of ^68^Ga-labelled tracers showed radiochemically pure and stable products at least four hours from end-of-synthesis.

## 1. Introduction

Gallium-68 (^68^Ga) is a positron-emitting radioisotope with a half-life of 68 min. This relatively short half-life is suitable for positron emission tomography (PET) diagnostics when radiolabeling small molecules with fast pharmacokinetics [1,2].

The ^68^Ge/^68^Ga-generators are easy to use but do suffer from several drawbacks. The small amount of radioactivity that can be eluted (e.g., ~1.5 GBq from a new generator) requires multiple generators to scale up radiopharmaceutical production. After elution, several hours are needed for ^68^Ga ingrowth before satisfactory radioactivity levels can be eluted again. This means the generator may be used for radiolabeling 2–3 times a day during normal working hours. Additionally, decay of the parent ^68^Ge leads to elution of less and less ^68^Ga radioactivity over the generator lifespan. If 3–4 patient doses per batch are typically obtained when the generator is new, only 1–2 doses per batch are produced toward the end of its use. The worldwide demand for ^68^Ga-based radiopharmaceuticals is constantly increasing, particularly since the successful introduction of theranostics, in which ^68^Ga is the diagnostic radionuclide. Although the commercial production of generators has recently expanded, there is still an unmet need for ^68^Ga, with long delivery times and relatively high pricing. Another drawback is the need to store expired generators for several years before destruction due to the 271-day half-life of germanium-68 (^68^Ge).

The approval of ^68^Ge/^68^Ga-generators has tremendously facilitated the clinical implementation of several ^68^Ga-labelled tracers in the last five years. The availability of ^68^Ge/^68^Ga-generators has had undoubted importance for the development of new radiopharmaceuticals for preclinical applications to clinical implementation, as well as for enabling hospitals without access to a cyclotron to produce their own PET radiopharmaceuticals [2,3].

Altogether, these limiting factors have driven the development of alternatives to ^68^Ge/^68^Ga-generators to improve the availability of ^68^Ga [4]. The ability to produce ^68^Ga with a low-energy cyclotron is an important development and has recently been clinically implemented [5].

The cyclotron production of ^68^Ga is feasible using either liquid or solid targets. In liquid-target productions, a solution of enriched ^68^Zn salt is irradiated to produce the desired radiometal via the ^68^Zn(p,n)^68^Ga reaction [6,7]. The zinc solution to be irradiated is conveniently transferred through transfer lines to the target holder before irradiation and also after irradiation to the synthesis hot cell for purification and radiolabeling in the radiopharmaceutical production. The yields of radiopharmaceuticals produced from liquid target ^68^Ga are similar or slightly increased compared to those using a ^68^Ge/^68^Ga-generator.

Production of ^68^Ga by the ^68^Zn(p,n)^68^Ga reaction using solid-target systems on low-energy medical cyclotrons [8,9,10] has yielded the highest radioactivity, up to 370 GBq [11]. In both liquid- and solid-target productions, it is of critical importance to separate ^68^Ga from the irradiated ^68^Zn and other metal ions. Many separation techniques have been suggested [12]. Incompletely removed metal ion impurities compete with Ga^3+^ in the complexation with different chelators, which negatively affects the radiolabeling yields. In addition to the zinc that must be removed, predominantly, the metal ion of concern is the trivalent Fe^3+^ [13], which has a higher stability constant (log K_ML_) for the chelator 1,4,7,10-tetraazacyclododecane-1,4,7,10-acetic acid (DOTA), for example, than Ga^3+^ [14,15].

Our group has recently developed a solid-target ^68^Ga production purification sequence based on double anion exchange Uranium and TEtraValents Actinides (UTEVA^®^) resin columns, washed in an effective last step with hydrochloric acid (HCl) (2.5 N) to minimize the content of Zn^2+^ (target material) ions remaining in the ^68^GaCl_3_ eluate [13]. All quality requirements, according to the European Pharmacopoeia monograph for cyclotron produced ^68^Ga [16], were fulfilled. An apparent molar activity (AMA) of 86 ± 22 GBq/µmol (*n* = 3), determined by DOTA titrations, was achieved. The content of Zn in the eluate (Zn to activity ratio) was satisfying, setting the shelf-life of the ^68^GaCl_3_ eluate to 7.7 h. The limiting factor was the content of Fe in the eluate (Fe to activity ratio), which set the final shelf-life of the ^68^GaCl_3_ eluate to 6.4 h. However, when using this ^68^GaCl_3_ eluate for radiopharmaceutical productions of [^68^Ga]Ga-DOTATOC or [^68^Ga]Ga-FAPI-46, an RCY of only approximately 25% were obtained, with a 40 or 50 µg precursor, respectively. From 10 GBq ^68^GaCl_3_ eluate, 2.5 GBq product was obtained. Although this means a three-fold higher product activity compared to generator-produced syntheses, there is still a large fraction of radioactivity that is lost during the synthesis. This prompted us to make further improvements to increase the RCY.

We hypothesized that decreasing the amount of Fe^3+^ in the cyclotron-produced ^68^GaCl_3_ eluate would increase the RCY, and consequently, the AMA.

**Figure 1:**
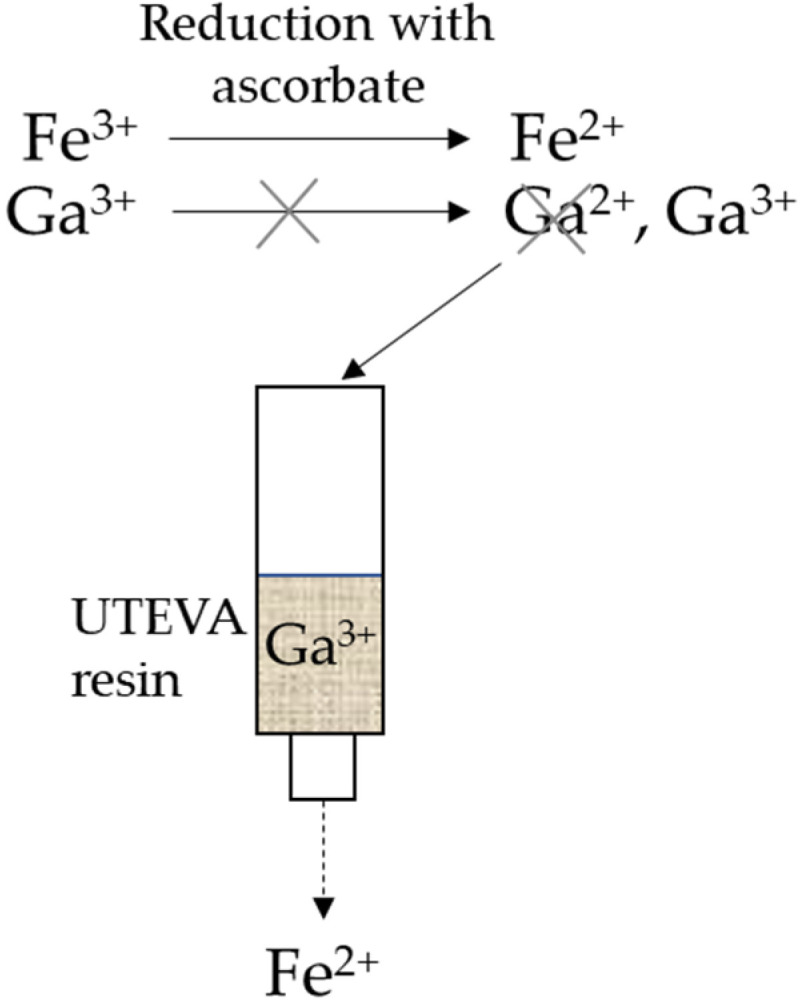
Fe^3+^ is reduced to Fe^2+^ by sodium ascorbate, decreasing the stability constant (log K_ML_) to the UTEVA resin. Ga^3+^ is not reduced by sodium ascorbate.

It is important to take into account that the metal ions are in constant equilibrium with the surrounding negatively charged counter ions and water molecules that act as ligands and form metal complexes. The speciation of the metal complexes is of crucial importance due to the charge and electrostatic interactions with the surrounding environment. High concentrations of chloride, and low pH, favor the formation of negatively charged complexes, such as [FeCl_4_]^−^ and [GaCl_4_]^−^. The negatively charged complexes follow the HCl concentration and the distribution coefficients of the metal ions for the UTEVA resin [17,18,19]. The active part of the UTEVA resin consists of a neutral dipentyl pentylphosphonate complexing ligand for the metal ion [17]. The reduction potential of Fe^3+^ to Fe^2+^ at a low pH is around +0.8 V and of Ga^3+^ to Ga^2+^ at around −0.6 V [20,21]. The oxidation potential of ascorbic acid at low pH is around −0.3 V [22]. Due to the lower reduction potential of Ga^3+^, which is lower than the oxidation potential of ascorbic acid, the result is a reduction of Fe^3+^ to Fe^2+^, while gallium is kept in the form of Ga^3+^. See Figure 1 below.

The aim of this work was to improve the purification methodology of the solid target production of ^68^Ga. By adding ascorbate to the purification steps, the level of Fe^3+^ in the ^68^GaCl_3_ eluate was significantly decreased; thus, enabling high-yield radiolabeling of clinically relevant DOTA-based tracers, such as DOTATOC and FAPI-46.

## 2. Materials and Methods

### 2.1. ^68^Ga Production and Purification Modification with Added Ascorbate

**Figure 2:**
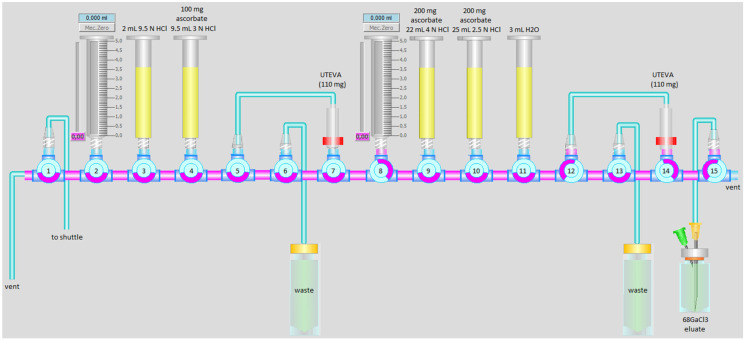
Schematic illustration of the automated protocol used for the separation of ^68^Ga from enriched ^68^Zn, using a cassette-based Taddeo PRF module (Comecer). To decrease the Fe^3+^ impurity, sodium ascorbate was added in position 4 (100 mg), position 9 (200 mg), and position 10 (200 mg).

^68^GaCl_3_ was produced via the ^68^Zn(p,n)^68^Ga and purified according to our previous method [13], except with an addition of 500 mg of sodium ascorbate (Apotekets Produktion och Laboratorier (APL), Stockholm, Sweden), divided between the HCl dilution and wash solutions as shown in Figure 2. In short, 110 mg ^68^Zn enriched (98.7 ± 0.2%) foil (Isoflex, San Francisco, CA, USA) was pneumatically transferred using a transfer module (Comecer EDS) to the cyclotron’s irradiation station (GE Healthcare, Uppsala, Sweden, PETtrace 800 and Comecer PTS). Irradiation was performed with a proton beam current of 25 µA for 68 min. Dissolution and separation were fully automated using a cassette-based Taddeo PRF module (Comecer, Castel Bolognese, Italy), and all materials and acids used were of metal-free quality, as stated in [13]. The RNP of the eluate was determined by gamma spectroscopy using a high-purity germanium detector (Canberra with Cryo-Cycle II Hybrid Cryostat), radionuclidic identity was determined by half-life measurement using a dose calibrator (Capintec CRC-55tR, LabLogic, Sheffield, United Kingdom), as described in [13].

### 2.2. Colorimetric Test of Iron Content and ICP-MS Measurements

To investigate that iron was eliminated to a larger extent when reduced to Fe^2+^ using ascorbate in the UTEVA resin purification method, we first performed a cold colorimetric measurement of the content of iron present in the rinses with or without the addition of ascorbate. The UTEVA resin (110 mg) was loaded with Fe^3+^ (Fe(III)Cl_3_, Sigma-Aldrich, Stockholm, Sweden), rinsed, and eluted in a fashion comparable to that used in the ^68^Ga purification of this study. The UTEVA resin was conditioned with HCl (4 N, 4 mL), 10/20/30 µg Fe^3+^ in HCl (4 N, 2 mL) (with or without 10 mg/mL ascorbate) was loaded and trapped on the resin, following rinses with HCl (4 N, 10 mL) (with or without 10 mg/mL ascorbate) and HCl (2.5 N, 8 mL) (with or without 10 mg/mL ascorbate), and lastly dried with 20 mL of air. The resin was then eluted using 1 mL of water (TraceSelect, Honeywell, Seetze, Germany), and the eluate was collected for analysis of iron content using an iron colorimetric test (MColortest, part no. 1.14759.0001, Merck, Darmstadt, Germany). The *p*-values were calculated using the Student’s *t*-test in Excel (Microsoft^®^ Excel^®^ for Microsoft 365MSO); *p* < 0.05 was considered statistically significant.

^68^GaCl_3_ eluate from one ^68^Ga solid-target cyclotron production with added ascorbate, and one without ascorbate, were analyzed by Inductively Coupled Plasma-Mass Spectrometry (ICP-MS) externally (ALS, Umeå, Sweden). The analysis included the following metal ions, Zn (calibrated for ^68^Zn instead of natZn), Fe, Ga, Al, Cd, Cu, Ge, Mo, Ni, Pb, Pt, and Ti.

### 2.3. Chelator Titrations and AMA Determination

AMA of the cyclotron-produced ^68^GaCl_3_ eluate was determined on 50 µL (5% of total eluate volume) by titration with the chelators DOTA (Sigma-Aldrich), (1,4,7-triazonane-1,4,7-triyl) triacetic acid (NOTA) (CheMatech, Dijon, France), and *N,N′*-Di(2-hydroxybenzyl)ethylenediamine-*N,N′*-diacetic acid monohydrochloride hydrate (HBED) (STEM Chemicals Inc., Bischheim, France). Chelator solutions were prepared in serial dilutions. Ranges of chelators labeled in the titrations, when ascorbate was used in the purification: DOTA 3.1 pmol–0.1 nmol, NOTA and HBED 1.2 pmol–0.05 nmol, and when ascorbate was not used in the purification: DOTA 31.2 pmol–1.0 nmol, NOTA and HBED 15.5 pmol–0.5 nmol. The ^68^GaCl_3_ solution was adjusted to pH 4.0 using sodium acetate buffer (~1:10 acetate buffer solution pH 4.6 (Honeywell Fluka, Steinheim, Germany) in TraceSelect Water (Honeywell) pH adjusted with HCl (Honeywell)) to a final volume of 600 µL in each vial. The vials were incubated at 95 °C, 550 rpm for 15 min (Eppendorf (ThermoMixer C)). The AMA was analyzed by measuring the labeling efficiency of each vial. Analysis of labeling efficiency (incorporation of ^68^Ga in DOTA, NOTA, and HBED) was performed by radio-thin layer chromatography using iTLC-SG-strip (Agilent, Folsom, CA, USA) as stationary phase, eluted in ammonium acetate 1 M (Sigma-Aldrich): methanol (Merck) 1:1 as mobile phase. In this analysis, free ^68^Ga stayed at the origin (Retardation factor, Rf~0–0.1) while complexed ^68^Ga-DOTA, ^68^Ga-NOTA, and ^68^Ga-HBED migrated (Rf~0.9–1.0). Radioactivity in the strips was detected by a TLC-scanner (AR-2000, Eckert & Ziegler, Berlin, Germany), and analysis was performed using the software WinScan 3.0 (Eckert & Ziegler). Labeling efficiency was plotted as a function of DOTA, NOTA, and HBED chelator mass (µmol). AMA was calculated by the equation of the line and determined as 50% incorporation and by dividing these values by two, as suggested earlier [23]. The values were decay-corrected to the end of ^68^GaCl_3_ eluate purification (EOP).

### 2.4. Synthesis of [^68^Ga]Ga-FAPI-46 and [^68^Ga]Ga-DOTATOC

From each cyclotron production of ^68^GaCl_3_ (total volume of ~1 mL), 50 µL of the eluate was used for chelator titration as described above. The remainder of the radioactivity (~11 GBq) was used for each radiopharmaceutical synthesis.

**Figure 3:**
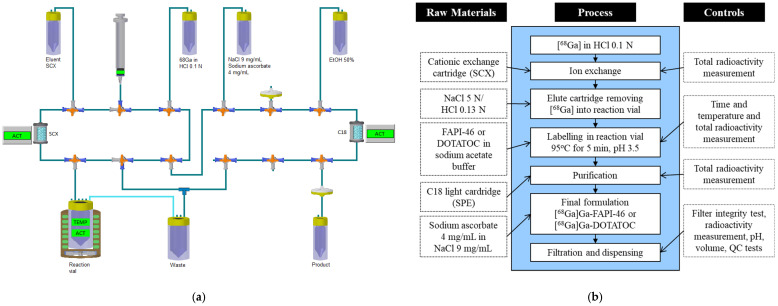
(**a**) Schematic illustration for the synthesis of [^68^Ga]Ga-FAPI-46 or [^68^Ga]Ga-DOTATOC using Modular-Lab Pharmtracer (Eckert and Ziegler). (**b**) Flow diagram of the GMP-compliant synthesis of ^68^Ga-based radiopharmaceuticals using Modular-Lab Pharmtracer synthesis module.

Automated radiosynthesis was performed on an Eckert & Ziegler Modular-Lab PharmTracer synthesis module using the Modular-Lab software (Eckert & Ziegler). See Figure 3 for a schematic flow diagram of the synthesis. All materials used for radiolabeling were of GMP grade and metal-free quality if not otherwise stated. All buffer kits and hardware kits (synthesis cassettes) for the syntheses were purchased from Eckert & Ziegler.

The ^68^GaCl_3_ eluate was diluted to 4–5 mL with 0.1 N HCl (Eckert & Ziegler) to minimize activity losses in the synthesis cassette-connected eluate transfer tube. The reaction vessel was prepared to contain 50 µg of FAPI-46 precursor (Sofie Biosciences, Totowa, NJ, USA) or 40 µg of DOTATOC precursor (ABX, advanced biochemical compounds, Radeberg, Germany) and buffer solution (54 mg sodium acetate trihydrate, 18 µL 30% HCl, 8 µL glacial acetic acid, 2.4 mL TraceSelect water, and 0.2 mL ethanol). The diluted ^68^GaCl_3_ eluate was transferred to the synthesis unit and trapped on a cationic exchange cartridge (SCX in the synthesis scheme, Figure 3a) and eluted into the reaction vial with 0.7 mL of sodium chloride (NaCl) 5 N/HCl 0.13 N. The final volume of the reaction mixture was 3.3 mL, pH 3.5. The labeling reaction mixture was heated to 95 °C for 5 min. After the end of the labeling, the crude product was diluted with 2 mL of 4 mg/mL sodium ascorbate in 9 mg/mL NaCl and trapped on a reversed-phase solid-phase extraction (SPE) cartridge (C18 in the synthesis scheme, Figure 3a). The SPE was rinsed to waste using 4 mL of 4 mg/mL sodium ascorbate in 9 mg/mL NaCl to remove any remaining free ^68^Ga ions in the system. The trapped product was then eluted from the SPE, using 1.2 mL of ethanol/water 1:1, through a 0.22 µm sterile filter (Millex-GV, Merck Millipore, Darmstadt, Germany) into the product vial. The product ([^68^Ga]Ga-FAPI-46 or [^68^Ga]Ga-DOTATOC) was lastly diluted with 4 mg/mL sodium ascorbate (APL, Sweden), as a radiolytic stabilizer, in 9 mg/mL NaCl to a final formulation volume of approximately 9.5 mL. From the addition of eluate to the finished product, the time required was 17 min. Radiosynthesis of these products using generator-based ^68^GaCl_3_ eluate was performed in the same way, using eluate from a GalliaPharm generator (Eckert & Ziegler) or a GalliAd generator (IRE ELiT, Fleurus, Belgium). The synthesis of [^68^Ga]Ga-DOTATOC was, however, performed without ascorbate as a stabilizer. A flow diagram of the syntheses is illustrated in Figure 3b.

### 2.5. Quality Control of [^68^Ga]Ga-FAPI-46 and [^68^Ga]Ga-DOTATOC

Full quality controls (QC) were performed for [^68^Ga]Ga-FAPI-46 and [^68^Ga]Ga-DOTATOC using qualified instruments if not otherwise stated. The QC attributes determined included the appearance by visual inspection and pH by pH strip (Merck, Darmstadt, Germany). The content of bacterial endotoxins was performed by chromogenic LAL-test method using Endosafe-Nextgen PTS (Charles River, Willmington, MA, USA), and the filter integrity was tested by a bubble point tester (DM Automation, Sweden or an in-house built, qualified bubble point tester).

The radiochemical purity (RCP), chemical purity, as well as radiochemical stability were measured by analytical radio-high performance liquid chromatography (radio-HPLC). Two different HPLC systems were used. The Agilent 1260 Infinity System is equipped with a quaternary pump, autosampler, and DAD UV detector (254 nm) as well as a FlowRAM 2”NaI/PMT radiodetector (LabLogic, Sheffield, United Kingdom) and the software Laura (LabLogic) was used for [^68^Ga]Ga-FAPI-46. Analysis was performed on an analytical column (Agilent Poroshell 120 EC-C18, 2.7 µm 4.6 × 100 mm) and a guard column (Poroshell 120 EC-C18 Fast guard, 3 × 5 mm, 2.7 µm). The mobile phase was a gradient composed of 50 mM phosphoric acid (H_3_PO_4_) and acetonitrile (CH_3_CN); a flow rate of 0.3 mL/min was used.

The Shimadzu HPLC system (Duisburg, Germany) is equipped with a binary pump, degasser (Biotech, Onsala, Sweden), manual injector (Rheodyne, Bensheim, Germany), and UV-VIS detector (220 nm), as well as a radiodetector (Bioscan, Washington, DC, USA) and the software Shimadzu LC Solution was used for [^68^Ga]Ga-DOTATOC. Analysis was performed using an analytical column (ACE 3-C18, 4.6 × 150 mm) and a guard column of the same material (3 µm). The mobile phase was a gradient composed of 0.1% TFA in CH_3_CN:H_2_O, and a flow rate of 0.6 mL/min was used.

The radiochemical impurities of ^68^Ga ions and ^68^Ga-colloids were determined with iTLC analysis using iTLC-SG strip (Agilent). The radioactivity was detected using a radio-TLC scanner, either Scan-RAM with a PS/PMT detector, equipped with the software Laura (LabLogic) or the Bioscan TLC scanner, equipped with the software Winscan (Bioscan). The mobile phase of 5 M ammonium acetate (Merck) and methanol (Merck) in a ratio of 25:75 was used for [^68^Ga]Ga-FAPI-46 while the mobile phase of 1 M ammonium acetate (Merck) and methanol (Merck) in a ratio 1:1 was used for [^68^Ga]Ga-DOTATOC. In these systems, Rf was ~0–0.2 for ^68^Ga-impurities, and Rf was ~0.6–1.0 for ^68^Ga-labeled products.

Ethanol levels in the products were analyzed using a gas chromatograph (GC model 6850 Agilent) equipped with a flame ionization detector, an Agilent Res-Solv column (30 m × 0.53 mm ID × 1.0 µm film), and an autoinjector. The GC method used a 2 µL injection volume, a split ratio of 1:80, and helium as a carrier gas. The temperature was programmed to 35 °C for 3.5 min after injection, ramped to 240 °C at a rate of 70 °C/min, held at 240 °C for 3 min, and cooled to 35 °C.

The stability (shelf-life) of ^68^Ga-labeled products was determined by analyzing the total radiochemical purity of the product with HPLC and iTLC as described above. Sterility tests were performed by direct inoculation by an external contractor (APL, Stockholm, Sweden).

## 3. Results

### 3.1. ^68^Ga Production and Radionuclidic Purity

Production of ^68^Ga (25 µA, 68 min, 110 mg ^68^Zn enriched foil (Isoflex), *n* = 8), yielded a ^68^GaCl_3_ eluate, corrected to the end of purification, of 76 ± 2%, corresponding to 11.3 ± 1.5 GBq. The yield was calculated as the product activity divided by the total amount of starting activity transferred to the purification cassette. The entire ^68^GaCl_3_ eluate obtained, except for 50 µL saved for titrations and other analyses, was used for individual radiolabelings (see Section 3.4). The radionuclidic purity of the eluate at the end of bombardment (EOB) was 99.94 ± 0.00% (*n* = 4) and the half-life was 68.7 ± 0.5 min (*n* = 4).

### 3.2. Verification of Iron Content after Addition of Ascorbate

**Figure 4:**
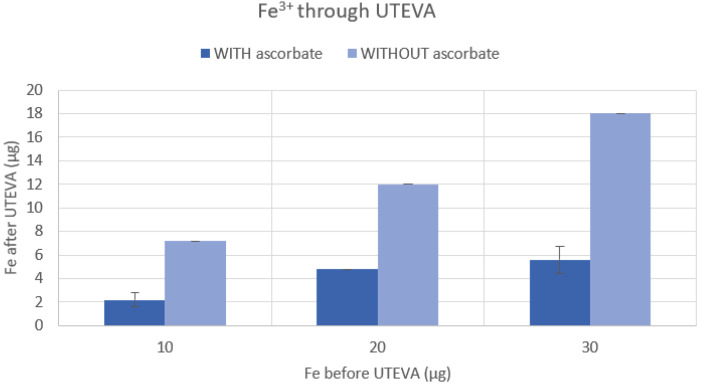
Comparison between the iron present in the eluate when the UTEVA resin was washed with sodium ascorbate added HCl (*n* = 3) and HCl without sodium ascorbate (*n* = 3). The Student’s *t*-test showed *p*-values < 0.001, which were considered significant. The iron concentrations were analyzed using a colorimetric test kit.

As illustrated in the colorimetric analysis in Figure 4, the addition of sodium ascorbate to the dilution and wash solutions significantly (*p* < 0.001) decreased the iron content compared to when no ascorbate was used. It was also evident that iron was efficiently removed by the UTEVA resin. ICP-MS analysis confirmed the efficiency of the removal of iron, as much as up to a 7-fold decrease of the iron content when sodium ascorbate was added in the purification process. ICP-MS analysis also showed that the only metal affected by the ascorbate addition was iron (see details in Table S1 in the Supplementary Materials). The affinity of Fe^2+^ complexes was interpreted to be lower on the UTEVA than for Fe^3+^ complexes, while the high affinity of Ga^3+^ complexes was retained.

### 3.3. Titrations with DOTA, NOTA, and HBED

**Figure 5:**
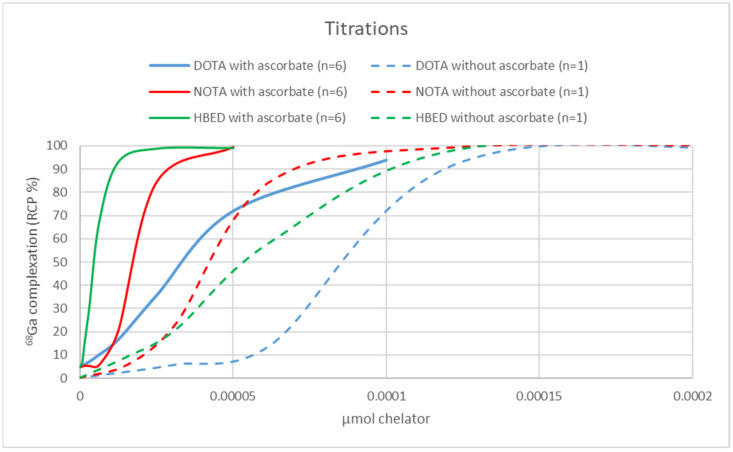
Comparison between DOTA, NOTA, and HBED titrations on ^68^GaCl_3_ productions with or without sodium ascorbate addition to the dilution and wash solutions in the purification process.

AMA results for titrations with DOTA, NOTA, and HBED are summarized in Table 1 and illustrated in Figure 5. For comparison, the AMA results for titrations with DOTA using generator-produced ^68^Ga from [13] are also shown in Table 1. The AMA values were 2-, 3- and 16-fold higher with ascorbate for DOTA, NOTA, and HBED, respectively, verifying a considerable improvement.

### 3.4. Synthesis and Quality Control of [^68^Ga]Ga-FAPI-46 and [^68^Ga]Ga-DOTATOC

Summaries of the syntheses and QC of [^68^Ga]Ga-FAPI-46 and [^68^Ga]Ga-DOTATOC syntheses are shown in Table 2 and Table 3, respectively. Data of the generator-produced syntheses of each radiopharmaceutical are based on the clinical GMP production for patients at Karolinska University Hospital. Initial test labeling of each peptide with the previous ^68^Ga production method without ascorbate gave an RCY of less than 25% (for example, for [^68^Ga]Ga-FAPI-46, the obtained batch activity was 2.45 GBq from a starting activity of 10.8 GBq). With this new ^68^Ga production method, including the addition of ascorbate, using the same amount of starting activity and precursor amounts, RCYs of 57% and 64% were obtained for [^68^Ga]Ga-FAPI-46 and [^68^Ga]Ga-DOTATOC, respectively. These RCYs were on the same level that is normally obtained with generator-produced ^68^Ga. The resulting radioactivity from each batch at EOS were 5.58 ± 0.35 GBq (*n* = 3) for [^68^Ga]Ga-FAPI-46 and 6.1 ± 1.3 GBq (*n* = 3) for [^68^Ga]Ga-DOTATOC. This batch radioactivity is 10 times higher than that normally obtained with generators, which were 0.58 ± 0.09 GBq (*n* = 4) for FAPI-26 and 0.61 ± 0.16 GBq (*n* = 86) for DOTATOC. Typical HPLC chromatograms of [^68^Ga]Ga-FAPI-46 and [^68^Ga]Ga-DOTATOC can be found in Figures S1 and S2, Supplementary Materials.

Quality control of [^68^Ga]Ga-DOTATOC and the product specifications were performed according to Ph. Eur. monograph (PA/PH/Exp. 14/T, monograph number 2482). QC and product specifications of [^68^Ga]Ga-FAPI-46 (EudraCT number 2020-002568-30) were based on the current draft of Ph. Eur. monograph of the ^68^Ga-radiolabeled product. Both were approved by the Medical Product Agency. Following these QC methods, both cyclotron-produced products fulfilled the specification criteria (see Table 2 and Table 3). The stability was evaluated up to 4 h EOS for both tracers, showing a total RCP of over 95%. Both products were stabilized with 4 mg/mL ascorbate; a longer stability than 4 h might have been obtained but was not measured.

## 4. Discussion

The feasibility to utilize all high out-put cyclotron-produced ^68^GaCl_3_ eluate for radiolabeling of DOTA-based tracers is highly dependent on the purity (i.e., content of competing metal ions impurities) of the ^68^Ga-eluate. Impurities may originate from the starting materials, i.e., dilution and wash solutions, tubing, and especially the target material (i.e., the ^68^Zn foil).

Low content of competing ions such as zinc and iron, especially the trivalent Fe^3+^, when for example, DOTA is used as a chelator in the radiolabeling, is of great importance. The importance of decreasing the content of Fe^3+^ in the ^68^GaCl_3_ eluate is the metal ions’ higher stability constant in association with the chelator, i.e., its’ ability to form stable complexes with the chelator [14,15].

In this study, we have demonstrated a straight-forward and improved purification approach to achieve a cyclotron solid target produced ^68^GaCl_3_ eluate with significantly lower levels of competing metal ions (i.e., Fe^3+^) by the addition of ascorbate. Ascorbate is a powerful antioxidant that is commonly used as a radiolytic stabilizer in radiopharmaceuticals [24,25]. Here, by utilizing sodium ascorbate’s predominant ability to reduce Fe^3+^ to Fe^2+^ and its inability to reduce Ga^3+^ to Ga^2+^, a more effective separation through the UTEVA resins is possible in the purification process. This enables the use of cyclotron-produced ^68^GaCl_3_ eluate for high-yield DOTA-chelate complexation with superior results.

AMA values, analyzed by titrations, were 2-, 3- and 16-fold higher for DOTA, NOTA, and HBED, respectively, verifying a considerable improvement. The difference in the AMA increase for the different chelators might possibly be explained by their binding stabilities and stability constants (log K_ML_) to Ga^3+^, Fe^3+^ and Fe^2+^, as further summarized in Table S2, in the Supplementary section. The incredible increase in AMA for HBED may be related to its high log K_ML_ to both Ga^3+^ and Fe^3+^. Notably, this purification approach has enabled high-yield DOTA-based radiopharmaceutical productions of 5.58 ± 0.35 GBq (*n* = 3) for [^68^Ga]Ga-FAPI-46 and 6.1 ± 1.3 GBq (*n* = 3) for [^68^Ga]Ga-DOTATOC.

High concentrations of competing metal ions may be, alternatively, compensated for by increasing the amounts of precursor used in the radiolabeling, as previously demonstrated [9,11]. For example, Tieu et al. [9] used 80 µg of DOTATATE precursor for labeling with 6.3 GBq ^68^GaCl_3_ and received 3.31 GBq [^68^Ga]Ga-DOTA-TATE (RCY = 70%, RCP = 68%). Thisgaard et al. [11] used 500 µg and received 3.22 GBq [^68^Ga]Ga-DOTA-TATE product. This approach will increase the produced radioactivity but will also lower the AMA. This could be problematic due to the restricted maximum peptide dose allowable for patient administration according to the European Pharmacopoeia (e.g., 50 µg of DOTATOC [26]). This would consequently limit the shelf life of the radiopharmaceutical product.

Currently, there is no clinical establishment defining the influence of AMA on the imaging utility of ^68^Ga-labeled tracers in oncological applications. It is, however, known from generator-produced batches that clinical imaging is feasible in the AMA ranges of 7–25 GBq/µmol. The impact of AMA has been closely investigated in some limited preclinical studies. Lin et al. reported that in vitro cell uptake and better contrast in in vivo preclinical imaging was seen with increasing AMA of [^68^Ga]Ga-PSMA-11 [27]. Increased in vitro cell uptake with AMA was also reported for the same radiotracer by Sanchez-Crespo et al. [28]. In a study by von Hacht et al., the low AMA of the DOTA-based ^68^Ga-labeling was resolved by preparative HPLC purification, thereby improving the detection of small metastases [29]. The level of AMA and its impact in diagnostics is an interesting and important aspect, which is made available also for DOTA-based ^68^Ga-labeled radiopharmaceuticals by the results from this present study.

It is of considerable interest to be able to utilize cyclotron-produced ^68^Ga eluate in kit preparations of ^68^Ga-based tracers, as more kits are elegantly prepared for one single vial compounding in which the eluate is directly added, thereby minimizing radiation exposure and handling. The high AMA cyclotron-produced ^68^Ga eluate obtained here warrants/can facilitate future kit preparation procedures.

## 5. Conclusions

In this study, we have demonstrated a purification approach to decrease the levels of competing metal ions (i.e., Fe^3+^) in cyclotron-produced ^68^GaCl_3_ eluate and enabled GMP-compliant high-yield DOTA-peptide synthesis of clinically relevant tracers. The ^68^GaCl_3_ eluate from the solid-target production may be used in its full volume for further DOTA-based ^68^Ga-labeling without compromising the radiochemical yields or the need of increasing the amounts of precursor. Titrations indicate that radiolabeling of NOTA- or HBED-based tracers may give even better yields. To our knowledge, the apparent molar activity, AMA in the range of 100 and 200 GBq/µmol obtained from the syntheses of the DOTA-based tracers, is so far the highest achieved using cyclotron-produced ^68^GaCl_3_ eluate based on our purification method.

## Figures and Tables

**Table 1:** Apparent molar activity, AMA, results from titrations with DOTA, NOTA, and HBED chelator. * For comparison, corresponding results with DOTA from the generator were taken from [13].

	^68^GaCl_3_ from Generator *	^68^GaCl_3_ from Cyclotron	
	No Ascorbate (*n* = 3)	No Ascorbate (*n* = 1)	with Ascorbate (*n* = 6)
Chelator	AMA (GBq/µmol)	AMA (GBq/µmol)	AMA (GBq/µmol)
DOTA	10 ± 3	209	491 ± 204
NOTA	Not analyzed	314	993 ± 405
HBED	Not analyzed	280	4480 ± 3060

**Table 2:** Summary of syntheses and QC of [^68^Ga]Ga-FAPI-46.

Parameter	Product Specification	Generator-Produced (*n* = 4 ± SD)	Cyclotron-Produced (*n* = 3 ± SD)
Start activity (GBq)	Not specified	0.99 ± 0.16	9.8 ± 0.26
Precursor mass (µg)	Not specified	50	50
Product activity/batch (GBq)	Not specified	0.58 ± 0.09	5.58 ± 0.35
Activity concentration (MBq/mL)	Not specified	60.5 ± 10.5	602 ± 45
Non-decay-corrected RCY (%)	Not specified	58.2 ± 3.2	57.0 ± 2.5
AMA (GBq/µmol)	Not specified	10.0 ± 1.7	98.8 ± 6.2
Appearance	Clear or slightly yellow. Free of particles	Conforms	Conforms
pH	4.0–8.0	5.3 ± 0	5.3 ± 0.3
Product identity [^68^Ga]Ga-FAPI-46	|Rt_RD_ –Rt_UV_| < 60 s	40 ± 9.8	31 ± 9.5
Total chemical purity (µg/mL)	≤10 µg/mL	≤10	≤10
Radiochemical impurity, B (%)	≤3%	0.2 ± 0.4	0.26 ± 0.05
Total radiochemical purity (%) RCP_Tot_ = (100 − B) × T	≥91%	98.3 ± 0.01	9 7.4 ± 0.81
Filter integrity (bar)	≥3.5 bar	4.2 ± 0.0 *	4.1 ± 0.06
Bacterial endotoxins (EU/mL)	<17.5 EU/mL	<5.0	<5.0
Ethanol (%)	<10%	6.4 ± 0.45	6.8 ± 0.26
Sterility	Sterile, 0 CFU	Sterile	Sterile **
Radiochemical stability ****	RCP_Tot_ ≥ 91%	95 ± 0.02	96 ± 1.5 ***

Abbreviations: RCY = radiochemical yield; Rt = retention time; RD = radiodetector; UV = ultraviolet detector; B = percentage of radioactivity due to impurity [^68^Ga]-ions or -colloids in TLC analysis; T = proportion of the radioactivity due to [^68^Ga]Ga-FAPI-46 in the HPLC analysis; RCP_Tot_ = Total radiochemical purity; * based on 3 batches; ** based on one batch; *** based on 2 batches; **** Stability was 3 h EOS for generator-produced and 4 h for cyclotron-produced.

**Table 3:** Summary of syntheses and QC of [^68^Ga]Ga-DOTATOC.

Parameter	Product Specification	Generator-Produced (*n* = 86 * ± SD)	Cyclotron-Produced (*n* = 3 ± SD)
Start activity (GBq)	Not specified	1.0 ± 0.2	9.3 ± 1.4
Precursor mass (µg)	Not specified	40	40
Product activity (GBq)	Not specified	0.6 ± 0.2	6.1 ± 1.3
Activity concentration (MBq/mL)	Not specified	70.7 ± 0.2	650 ± 124
Non-corrected RCY (%)	Not specified	60.9 ± 7.8	64.4 ± 4.7
AMA (GBq/µmol)	Not specified	21.7 ± 5.6	215.1 ± 44.8
Appearance	Clear or slightly yellow. Free of particles	Conforms	Conforms
pH	4.0–8.0	5.8 ± 0.4	5.5 ± 0.3
Product identity	|Rt_RD_–Rt_UV_| < 120 s	83 ± 7	42 ± 6
[^68^Ga] gallium ion on HPLC	≤2%	Not detected	Not detected
Edotreotide plus [^68^Ga] ^68^Ga-DOTATOC	≤5 µg/mL	≤5	≤5
Radiochemical impurity, B (%)	≤3%	0.81 ± 0.61	0.18 ± 0.16
Total radiochemical purity (%) RCP_Tot_ = (100 − B) × T	≥91%	98.6 ± 3.6	99.8 ± 0.2
Filter integrity (bar)	≥3.5 bar	4.0 ± 0.1 **	4.1 ± 0.2
Bacterial endotoxins (EU/mL)	<17.5 EU/mL	<5 **	<5
Ethanol (%)	<10%	6.49 ± 0.32 **	6.4 ± 0.2
Sterility	Sterile, 0 CFU	Sterile **	Sterile
Radiochemical stability (%) ***	RCP_Tot_ ≥ 91%	97.7	99.2 ± 0.1

Abbreviations: RCY = radiochemical yield; Rt = retention time; RD = radiodetector; UV = ultraviolet detector; B = percentage of radioactivity due to impurity [^68^Ga]-ions or -colloids in TLC analysis; T = proportion of the radioactivity due to [^68^Ga]Ga-DOTATOC in the HPLC analysis; RCP_Tot_ = Total radiochemical purity; * Based on clinical batches; ** Based on 10 batches; *** Stability was 3 h EOS for generator-produced based on one batch while 4 h EOS for cyclotron-produced.

## Data Availability

The data presented in this study are available on request to the corresponding authors.

## Supplementary Materials

The following are available online at https://www.mdpi.com/article/10.3390/biom11081118/s1, Figure S1: Representative HPLC chromatograms for the quality control of [^68^Ga]Ga-FAPI-46 where A. UV detection at 264 nm of the reference sample of Ga-FAPI-46 and B. Radiochromatogram of [^68^Ga]Ga-FAPI-46, Figure S2: Representative HPLC chromatograms for the quality control of [^68^Ga]Ga-DOTATOC, where A. UV detection at 220 nm of the reference sample of edotreotide and B. Radiochromatogram of [^68^Ga]Ga-DOTATOC sample, Table S1: ICP-MS analysis of cyclotron-produced ^68^GaCl_3_ eluate, Table S2: Comparison of the log stability constants, log K_ML_ for the different chelators.
