# Supplementary material for: Clinically Applicable Cyclotron-Produced Gallium-68 Gives High-Yield Radiolabeling of DOTA-Based Tracers
Source: Biomolecules. 2021 Jul 29;11(8):1118. doi: 10.3390/biom11081118 (PMC8393313; doi:10.3390/biom11081118)
Supplement: Supplementary file 1 [file biomolecules-11-01118-s001.zip › biomolecules-1301937-supplementary.pdf]

Clinically Applicable Cyclotron-Produced Gallium-68 Gives High-Yield Radiolabeling of DOTA-Based Tracers

Emma Jussing[1,2,*,†], Stefan Milton[1,2,†], Erik Samén[1,2], Mohammad Mahdi Moein[1,2], Lovisa Bylund[2], Rimma Axelsson[3,4], Jonathan Siikanen[1,3], Thuy A. Tran[1,2,*]

1 Department of Oncology and Pathology, Karolinska Institutet, SE-171 77 Stockholm, Sweden
2 Department of Radiopharmacy, Karolinska University Hospital, SE-171 76 Stockholm, Sweden
3 Department of Medical Radiation Physics and Nuclear Medicine, Karolinska University Hospital, SE-171 76 Stockholm, Sweden
4 Department of Clinical Science, Intervention and Technology, Karolinska Institutet, SE-171 77 Stockholm, Sweden

* Correspondence: emma.jussing@ki.se (E.J.) and thuy.tran@ki.se (T.A.T.)
† Equally contributed

Table S1: ICP-MS analysis of cyclotron-produced $^{68}GaCl_3$ eluate

Table S1: ICP-MS analysis of cyclotron-produced $^{68}GaCl_3$ eluate

Metal impurity	With ascorbate (n=1) μg/L	Without ascorbate (n = 1) μg/L
**Fe**	**305**	**2100**
Al	24.1	23.3
Cd	<0.04	<0.04
Cu	3.72	3.93
Ga	1.53	1.80
Ge	<0.5	<0.5
Mo	6.75	9.30
Ni	<1	<1
Pb	<0.2	0.229
Pt	0.108	0.145
Ti	<0.02	<0.02
Zn	345	327

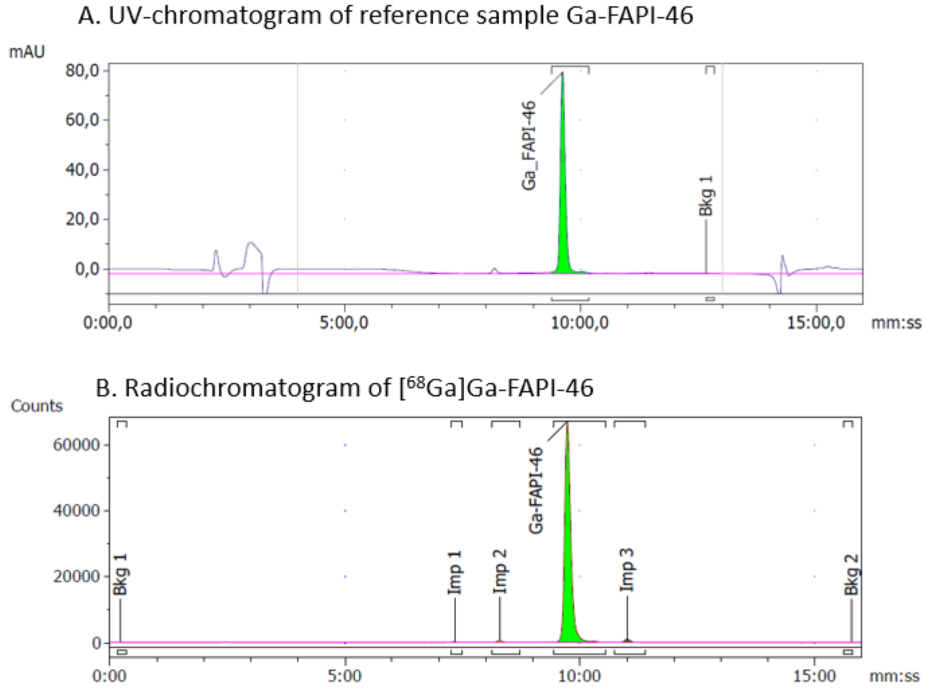

Figure S1: Representative HPLC chromatograms for the quality control of [^{68}Ga]Ga-FAPI-46 where A. UV detection at 264 nm of the reference sample of Ga-FAPI-46 and B. Radiochromatogram of [^{68}Ga]Ga-FAPI-46

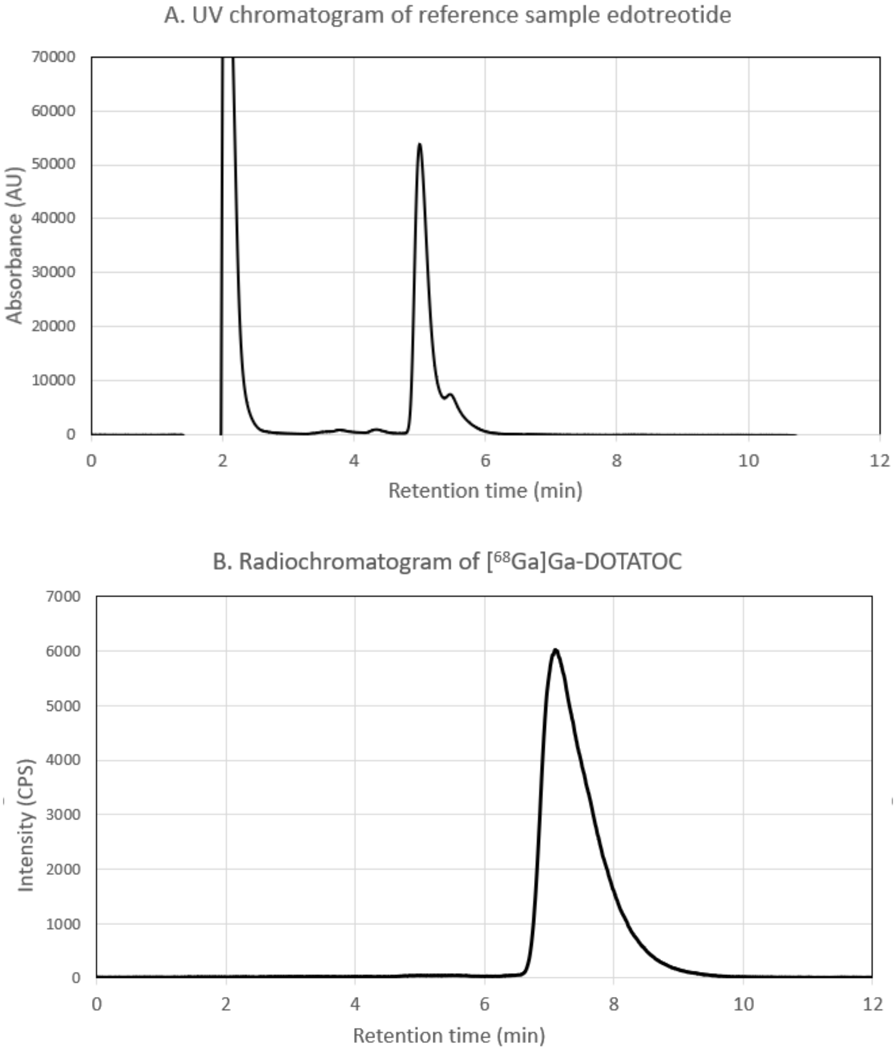

Figure S2: Representative HPLC chromatograms for the quality control of [^{68}Ga]Ga-DOTATOC, where A. UV detection at 220 nm of the reference sample of edotreotide and B. Radiochromatogram of [^{68}Ga]Ga-DOTATOC sample.

Table S2: Comparison of the log stability constants, log K_{ML} for the different chelators

Table S2. Comparison of the log stability constants, log K_{ML} for the different chelators				
**Chelator**	**Ga^{3+}**	**Fe^{3+}**	**Fe^{2+}**	**References**
DOTA	21.3 [a]	29.4 [a]	20.22 [b]	[a] [1]<br>[b] [2]
NOTA	31.0 [c]	28.3 [c]	NF*	[c] [3]
HBED	38.5 [d]	39.0 [d]	22.7 [b]	[b] [2]<br>[d] [4]

*NF = not found in the literature

References

1. Clarke, E.T.; Martell, A.E. Stabilities of trivalent metal ion complexes of the tetraacetate derivatives of 12-, 13- and 14-membered tetraazamacrocycles. *Inorganica Chimica Acta* **1991**, *190*, 37-46, doi:https://doi.org/10.1016/S0020-1693(00)80229-7.
2. Martell, A.E.; Motekaitis, R.J.; Chen, D.; Hancock, R.D.; McManus, D. Selection of new Fe(III)/Fe(II) chelating agents as catalysts for the oxidation of hydrogen sulfide to sulfur by air. *Canadian Journal of Chemistry* **1996**, *74*, 1872-1879, doi:10.1139/v96-210.
3. Clarke, E.T.; Martell, A.E. Stabilities of the Fe(III), Ga(III) and In(III) chelates of N,N′,N″-triazacyclononanetriacetic acid. *Inorganica Chimica Acta* **1991**, *181*, 273-280, doi:https://doi.org/10.1016/S0020-1693(00)86821-8.
4. Ma, R.; Motekaitis, R.J.; Martell, A.E. Stability of metal ion complexes of N,N′-bis(2-hydroxybenzyl)ethylenediamine-N,N′-diacetic acid. *Inorganica Chimica Acta* **1994**, *224*, 151-155, doi:https://doi.org/10.1016/0020-1693(94)04012-5.
